# Factors associated with anxiety and depression in patients with erectile dysfunction: a cross-sectional study

**DOI:** 10.1186/s40359-023-01074-w

**Published:** 2023-02-04

**Authors:** Yang Xiao, Tongjin Xie, Jingxuan Peng, Xun Zhou, Jinsong Long, Mohan Yang, Haiyan Zhu, Jianfu Yang

**Affiliations:** 1 Department of Urology, The Third Xiangya Hospital of Central South University, Changsha, China; 2 Department of Urology, Xiangya Hospital of Central South University, Changsha, China; 3 Department of Anesthesiology, The Third Xiangya Hospital of Central South University, Changsha, China

**Keywords:** Depression, Anxiety, Erectile dysfunction, SAS, SDS

## Abstract

**Background:**

Few studies have investigated factors associated with anxiety and depression among patients with erectile dysfunction (ED). This study aimed to investigate associated factors and the prevalence of anxiety and depression in this special group in China.

**Methods:**

Data from 511 patients with ED aged 18–60 years were collected between July 2021 and April 2022. The 5-item International Index of Erectile Function (IIEF-5) questionnaire, self-rating anxiety scale (SAS) and self-rating depression scale (SDS) were used to evaluate erectile function, anxiety and depression, respectively. Univariate analysis and multivariate linear regression analyses were used to explore the associated factors of depression and anxiety.

**Results:**

The prevalence of anxiety and depression among ED patients was 38.16% and 64.97%, respectively. The mean anxiety index score was 47.37 ± 6.69 points, and the mean depression index was 54.72 ± 9.10 points. Multiple linear regression analysis showed that worse ED, low education level, and smoking were positively associated with increased risk of anxiety and depression. In addition, younger age, longer onset time, and irregular sleep were positively associated with high risk of anxiety, and irregular exercise was associated with severe depression.

**Conclusions:**

The prevalence of depression and anxiety in ED patients is high, and the severity of ED, age, education level, smoking, onset time, regular sleep, and exercise were associated with anxiety or depression. Reversible risk factors should be avoided and individualized psychological support services are necessary for ED patients.

## Introduction

Erectile dysfunction (ED) is defined as the inability to achieve and maintain sufficient erection to allow for satisfactory sexual performance [1]. Epidemiological data have shown that ED is highly prevalence worldwide and is currently one of the most common sexual dysfunctions in men [2]. A previous study showed that the prevalence of ED varied from 37.2 to 48.6% in eight high burden countries [3]. In New Zealand, it was found that nearly a third of men aged 40–70 had ED but only 16% of them received a medical diagnosis and 22% are treated [4]. ED is described as a disrupted bio-psycho-social process involving the psychological, endocrine, vascular and nervous systems [5], which can significantly affect an individual's psychological well-being [6]. This suggests that the mental health of ED patients should be investigated and closely monitored.

Psychiatric disorder is strongly associated with erectile dysfunction, and their relationship seems to be bidirectional but not thoroughly studied [7–9]. A meta-analysis showed that depression increases the risk of ED and that ED also increases the risk of depression [10]. Sexual failure can lead to anxiety, increased fear, loss of confidence in sex, and lead to avoidance of sex, ultimately increasing the likelihood of future failure and creating a vicious circle [11]. A study in Greek found that 63.1% of ED patients had detectable mental illness, including 25.2% of depression [12]. They also found a positive correlation between the severity of depressive symptoms and the tendency of patients to discuss sex, but no significant correlation with the severity and duration of ED. Understanding the factors influencing the psychological response of ED patients can help identify high-risk groups of psychological disorders and formulate early individualized interventions.

Although some clinical studies have investigated the subject of sexual dysfunction, the association between psychiatric disorders and ED is not well characterized [13–15], especially no studies on the influencing factors. In addition, studies on ED may in terms of regions, periods, and measurement methods, thus it is necessary to analyze ED patients under different cultural, ethnic and socio-economic backgrounds.

Therefore, this research sought to investigate the associated factors and prevalence of anxiety and depression among ED patients in China. The findings will help to intervene reversible risk factors and formulate early individualized treatment for high-risk ED patients.

## Methods

### Study design

This was a questionnaire-based cross-sectional survey conducted in the andrology center of the Third Xiangya Hospital of Central South University in Hunan, China from July 2021 to April 2022. Using the PASS 15 software and with a confidence level of 0.95, an estimated prevalence rate of 30%, and error not exceeding 4%, sample size was calculated to be 502.

### Participants

Patients who complained of erectile dysfunction and visited the outpatient department were consecutively enrolled, and all participants were required to complete the questionnaire independently in a separate room after obtaining informed consent. Consultation was allowed when confused about any options, and the completed questionnaire would be checked by staff. A total of 550 respondents were recruited during this period, and 511 participants who met inclusion and did not meet the exclusion criteria were included in the study. The inclusion criteria were as follows: (1) men aged 18–60; (2) sexual life history over 6 months; (3) scores of the 5-item International Index of Erectile Function (IIEF-5) questionnaire are between 5 and 21. The exclusion criteria were as follows: (1) cognitive or communication disorders; (2) previous serious mental illness; (3) history of severe chronic diseases. All participants gave informed consent and could withdraw or interrupt from the study at any time. This study was approved by the IRB of The Third Xiangya Hospital of Central South University (date: 24/04/2019; number: 2019-S252) and registered in the Chinese Clinical Trial Registry (date: 05/09/2019; number: ChiCTR1900025700).

### Study questionnaire

The paper questionnaire consisted of three parts, and all scales in this study were Chinese version. The first part collected general information including living habits, age, education level, body mass index (BMI), occupation, sexual behavior among others. The second part contained the IIEF-5 questionnaire for evaluating erectile function, and the third part included self-rating anxiety scale (SAS) and self-rating depression scale (SDS) for anxiety and depression.

The IIEF-5 questionnaire is an effective tool used to detect the existence and severity of ED and has been linguistically validated in multilingual languages [16–18]. The Chinese version of IIEF-5 scale has been validated and extensively applied [19, 20]. Based on the Chinese version of IIEF-5 scale scores, the severity categories of ED was classified as follows: no ED (22–25 points), mild (17–21 points), mild to moderate (12–16 points), moderate (8–11 points) and severe (5–7 points).

SAS and SDS scales are standard assessment tools, whose validity and reliability have been examined in several studies [21, 22]. The SAS is a self-report measurement psychological scale developed by Zung in 1971 to evaluate anxiety [23]. It includes twenty items, five of which are reverse scores, and each item can be scored 1 to 4 points according the following options: 1 (no score or very limited score time), 2 (A small amount of time), 3 (for a long time) and 4 (most or all of the time). A SAS index score is the raw scores of all items multiplied by 1.25 to obtain the integer, which can be divided as follows: severe anxiety (≥ 70 points), moderate anxiety (60–69 points), mild anxiety (50–59 points) and no anxiety (< 50 points).

The SDS is a psychological scale established by Zung in 1965 to evaluate depression [24]. Similar to SAS, it also has 20 items, 10 of which are reverse scores. In the Chinese version of the SDS scales, a cut-off score of 53 points (a raw score of 42) has been recommended in the Chinese population [25], which has since been adopted by several Chinese studies [26, 27]. The SDS index score can be divided as follows: severe depression (≥ 73 points), moderate depression (63–72 points), mild depression (53–62 points) and no depression (< 53 points).

### Statistical analysis

Data were analyzed using the Statistical Product and Service Solutions (SPSS, version 26.0, IBM, Armonk, NY, USA). Frequencies were used to describe categorical variables, and means ± standard deviation (SD) were used to represent scale scores. The Kolmogorov–Smirnov test was used to test normal distribution. Independent sample t-test or single-factor analysis of variance (ANOVA) was used to compare the scores of depression and anxiety scales among different groups and multivariate linear regression analysis was used to identify associated factors of depression and anxiety. Categorical variables (hobbies and occupations) in the multivariate regression analysis were encoded as dummy variables. *P* < 0.05 was statistically significant.

## Results

### Demographic characteristics

A total of 550 respondents were recruited where 28 did not meet inclusion criteria and 11 met exclusion criteria. Finally, 511 males with ED were enrolled. The Cronbach alpha coefficient of the IIEF-5 questionnaire we used was 0.81, the SDS scale was 0.77, and the SAS scale was 0.66, showing sufficient internal consistency. Baseline characteristics of ED patients are detailed in Table 1. The mean age and IIEF-5 score was 33.79 ± 8.20 years and 12.76 ± 4.45 points, respectively. Approximately 35.23% had sought treatment for ED and 22.7% had different sexual partner, 49.51% were smokers, 35.62% were alcohol consumers, 58.32% had irregular sleep and 40.31% had no regular exercise. According to the IIEF-5 score, 14.29% of patients had severe ED, 27.40% had moderate ED, 35.81% had mild to moderate ED and 22.50% had mild ED.

**Table 1:** Demographic characteristics and Univariate analysis of anxiety and depression in 511 ED patients

Characteristic	Case (%)	Anxiety score (mean ± SD)	*P*-value	Depression score (mean ± SD)	*P*-value
*Age (years)*					
≤ 25	80 (15.66%)	50.03 ± 7.99	< 0.001^**^	55.15 ± 9.86	0.726
≤ 35	249 (48.73%)	47.06 ± 5.80		54.25 ± 9.14	
≤ 45	129 (25.24%)	46.91 ± 6.44		55.11 ± 8.49	
> 45	53 (10.37%)	45.91 ± 8.13		55.34 ± 9.28	
*BMI*					
< 18.5	10 (1.96%)	50.00 ± 5.60	0.634	57.10 ± 8.43	0.739
< 24	223 (43.64%)	47.26 ± 7.01		54.46 ± 9.16	
< 28	197 (38.55%)	47.26 ± 6.45		55.05 ± 9.04	
≥ 28	81 (15.85%)	47.57 ± 6.56		54.35 ± 9.24	
*Education level*					
Junior high school or below	98 (19.18%)	49.52 ± 6.52	< 0.001^**^	58.84 ± 7.01	< 0.001^**^
Senior high school	106 (20.74%)	47.97 ± 6.22		56.78 ± 7.35	
Junior college	132 (25.83%)	47.36 ± 6.61		54.67 ± 9.64	
Undergraduate or above	175 (34.25%)	45.80 ± 6.79		51.21 ± 9.39	
*Occupation*					
Official	75 (14.68%)	45.79 ± 6.94	0.031^*^	51.23 ± 9.50	< 0.001^**^
Corporate Employee	133 (26.03%)	47.34 ± 7.17		54.02 ± 10.04	
Self-employed or Freelance	104 (20.35%)	46.57 ± 6.29		55.50 ± 8.82	
Farmers	77 (15.07%)	48.66 ± 6.28		57.95 ± 6.00	
Others	122 (23.87%)	48.23 ± 6.40		54.94 ± 8.92	
*Hobbies*					
Computer/Video Games	211 (41.29%)	48.14 ± 6.66	0.011^*^	55.62 ± 8.97	0.039^*^
Ball game	78 (15.26%)	46.94 ± 6.82		54.46 ± 9.12	
Fitness/Jogging	115 (22.50%)	45.70 ± 6.42		52.70 ± 9.28	
Others	107 (20.94%)	47.95 ± 6.70		55.32 ± 8.90	
*Smoking*					
No	258 (50.49%)	46.33 ± 7.06	< 0.001^**^	52.79 ± 9.65	< 0.001^**^
Yes	253 (49.51%)	48.42 ± 6.13		56.69 ± 8.05	
*Alcohol drinking*					
No	329 (64.38%)	46.96 ± 6.94	0.068	54.51 ± 9.44	0.474
Yes	182 (35.62%)	48.09 ± 6.17		55.11 ± 8.45	
*Coffee drinking*					
No	443 (86.69%)	47.43 ± 6.58	0.601	55.14 ± 8.85	0.007^*^
Yes	68 (13.31%)	46.97 ± 7.41		51.97 ± 10.20	
*Regular sleep*					
No	298 (58.32%)	48.24 ± 6.78	< 0.001^**^	55.15 ± 9.25	0.211
Yes	213 (41.68%)	46.15 ± 6.39		54.13 ± 8.86	
*Regular exercise*					
No	206 (40.31%)	47.94 ± 6.49	0.113	56.80 ± 8.82	< 0.001^**^
Yes	305 (59.69%)	46.98 ± 6.81		53.32 ± 9.02	
*Treatment history*					
No	331 (64.77%)	46.67 ± 6.78	0.001^*^	54.09 ± 9.43	0.032^*^
Yes	180 (35.23%)	48.64 ± 6.36		55.89 ± 8.35	
*Regular sexual partner*					
No	116 (22.70%)	48.88 ± 6.72	0.005^*^	54.92 ± 8.87	0.788
Yes	395 (77.30%)	46.92 ± 6.63		54.66 ± 9.17	
*Onset time*					
< 12 moths	266 (52.05%)	46.37 ± 6.32	< 0.001^**^	54.35 ± 9.49	0.335
≥ 12 moths	245 (47.95%)	48.44 ± 6.93		55.13 ± 8.65	
*Frequency of intercourse*					
0–1 times (per week)	370 (72.41%)	47.43 ± 6.77	0.945	54.38 ± 9.22	0.180
2–3 times (per week)	117 (22.90%)	47.20 ± 6.65		56.05 ± 8.37	
≥ 4 times (per week)	24 (4.70%)	47.25 ± 5.87		53.54 ± 10.35	
*Severity of ED (IIEF-5)*					
Mild	115 (22.50%)	45.37 ± 6.75	< 0.001^**^	52.78 ± 10.65	0.032^*^
Mild to moderate	183 (35.81%)	47.23 ± 6.24		54.77 ± 8.35	
Moderate	140 (27.40%)	47.97 ± 6.83		55.29 ± 8.88	
Severe	73 (14.29%)	49.68 ± 6.66		56.56 ± 8.26	

Data were assessed with independent sample t-test or single-factor analysis of variance. *BMI* Body mass index, *ED* Erectile dysfunction, *IIEF-5* 5-item International Index of Erectile Function. ** *p* < 0.001, * *p* < 0.05

### Anxiety and depression

The severity categories of depression and anxiety are shown in Table 2. The prevalence of anxiety was 38.16% and the mean anxiety index score was 47.37 ± 6.69 points. The prevalence of depression was 64.97% and the mean depression index score was 54.72 ± 9.10 points. About 31.51% of ED patients had both anxiety and depression.

**Table 2:** The severity categories of anxiety and depression among ED patients

Severity of the symptoms	Anxiety *N* (%)	Depression *N* (%)
None	316 (61.84%)	179 (35.03%)
Mild	169 (33.07%)	247 (48.34%)
Moderate	26 (5.09%)	85 (16.63%)
Severe	0 (0%)	0 (0%)
Both anxiety and depression	161 (31.51%)	161 (31.51%)

Data are expressed as numbers (*N*) and percentages (%)

### Univariate analysis

Univariate analysis was performed to assess the association between depression and anxiety and demographic variables, including age, BMI, education level, occupation, hobbies, smoking, alcohol drinking, coffee drinking, regular sleep, regular exercise, treatment history, regular sexual partner, onset time, frequency of intercourse, and severity of ED (Table 1). The factors associated with depression and anxiety are not always the same. The results revealed that age (*p* < 0.001), education level (*p* < 0.001), occupation (*p* = 0.031), hobbies (*p* = 0.011), smoking (p < 0.001), regular sleep (*p* < 0.001), treatment history (*p* < 0.001), regular sexual partner (*p* = 0.005), onset time (*p* < 0.001), and severity of ED (*p* < 0.001) were significantly associated with anxiety, whereas education level (*p* < 0.001), occupation (p < 0.001), hobbies (*p* = 0.039), smoking (*p* < 0.001), coffee drinking (*p* = 0.007), regular exercise (*p* < 0.001), treatment history (*p* = 0.032), and severity of ED (*p* = 0.032) were significantly associated with depression.

### Multiple linear regression analysis

Factors with statistical significance in the univariate analysis were used as independent variables and anxiety or depression index scores were used as dependent variables for multivariate analysis. Results showed that a worsening ED (49.68 ± 6.66), low education level (49.52 ± 6.52), smoking (48.42 ± 6.13), younger age (50.03 ± 7.99), longer onset time (48.44 ± 6.93), and irregular sleep (48.24 ± 6.78) were positively associated with a high risk of anxiety (Table 3*R*^2^ = 0.17). In addition, worse ED (56.56 ± 8.26), low education level (58.84 ± 7.01), smoking (56.69 ± 8.05), and irregular exercise (56.80 ± 8.82) was associated with increased risk of depression (Table 4*R*^2^ = 0.169).

**Table 3:** The multiple linear regression of the anxiety in 511 ED patients

Independent variables	B	SE	95% CI		*P*-value
			Lower	Upper	
Severity of ED (IIEF-5)	1.11	0.291	0.539	1.681	< 0.001^**^
Education level	− 1.043	0.305	− 1.643	− 0.444	< 0.001^**^
Age	− 1.179	0.359	− 1.883	− 0.474	0.001^*^
Smoking	1.33	0.578	0.194	2.467	0.022^*^
Onset time	1.162	0.574	0.035	2.289	0.043^*^
Regular sleep	− 1.614	0.581	− 2.755	− 0.473	0.006^*^
Regular sexual partner	− 0.704	0.687	− 2.054	0.646	0.306
Treatment history	0.552	0.601	− 0.629	1.733	0.359
*Occupation*					
Official	Ref	Ref	Ref	Ref	Ref
Corporate Employee	− 0.014	1.018	− 2.014	1.985	0.989
Self-employed or Freelance	1.246	0.914	− 0.55	3.042	0.174
Farmers	1.404	1.166	− 0.886	3.694	0.229
Others	1.513	0.956	− 0.365	3.39	0.114
*Hobbies*					
Computer/Video Games	Ref	Ref	Ref	Ref	Ref
Ball game	− 1.13	0.753	− 2.611	0.35	0.134
Fitness/Jogging	0.315	0.843	− 1.34	1.971	0.708
Others	0.402	0.756	− 1.084	1.888	0.595

Linear regression analysis was used to identify associated factors of anxiety. *CI* Confidence interval. Ref: Reference category. ** *p* < 0.001. * *p* < 0.05

**Table 4:** The multiple linear regression of the depression in 511 ED patients

Independent variables	B	SE	95% CI		*P*-value
			Lower	Upper	
Severity of ED (IIEF-5)	0.984	0.391	0.216	1.752	0.012^*^
Education level	− 1.843	0.412	− 2.652	− 1.033	< 0.001^**^
Smoking	3.011	0.78	1.478	4.545	< 0.001^**^
Regular exercise	− 1.697	0.812	− 3.292	− 0.101	0.037^*^
Coffee Drinking	− 2.055	1.129	− 4.272	0.163	0.069
Treatment history	0.208	0.806	− 1.376	1.792	0.796
*Occupation*					
Official	Ref	Ref	Ref	Ref	Ref
Corporate Employee	1.453	1.374	− 1.247	4.153	0.291
Self-employed or Freelance	1.953	1.232	− 0.466	4.373	0.113
Farmers	2.4	1.571	− 0.687	5.487	0.127
Others	1.157	1.285	− 1.368	3.683	0.368
*Hobbies*					
Computer/Video Games	Ref	Ref	Ref	Ref	Ref
Ball game	− 1.805	1.027	− 3.822	0.213	0.079
Fitness/Jogging	0.825	1.146	− 1.427	3.077	0.472
Others	− 0.954	1.007	− 2.933	1.025	0.344

Linear regression analysis was used to identify associated factors of depression. *CI* Confidence interval. *Ref* Reference category. ** *p* < 0.001. * *p* < 0.05

## Discussion

This research identified factors associated with anxiety and depression among ED patients in China. About 38.16% and 64.97% of ED patients had anxiety and depression respectively, whereas 31.51% of ED patients had both anxiety and depression; and most of the symptoms were mild. The prevalence of anxiety and depression is higher than that reported in a previous study in Greece (anxiety disorders in 11.7% and depressive disorders in 25.2%) and Japan (anxiety was 7.7% -29.1% and depression was 13.6% -32.1% in different age groups) [12, 28]. This difference may be caused by geographical, cultural or socio-economic differences, especially given that it was conducted after the COVID-19 disease outbreak. Anxiety symptoms in the general population have nearly doubled to 24% compared to before the COVID-19 outbreak [29]; anxiety and depression have been reported to be major psychological challenges during the COVID-19 pandemic [30–32].

In our study, more than a third of ED patients had moderate or severe ED, and multivariate linear regression analysis demonstrated that anxiety and depression were significantly positively associated with ED severity. Patients with worse ED (a lower IIEF-5 score) are more likely to develop anxiety or depression. This is similar to findings from some previous studies [33, 34], but differs from one European report [12]. The latter study found that depressive symptoms were not associated with ED severity, although they enrolled a small sample size. Moreover, the mechanism linking ED and psychological problems has not been well studied. ED patients tend to have low positive emotions and negative expectations about their sexual performance. This mental state distracts their attention from erotic signals, leading to inhibition of genital arousal. As a consequence, some men will avoid sex, which worsens their initial negative sex-related effect [35]. Therefore, we speculate that men with severe ED will experience higher stigma, weakness, and fear, all of which negatively affect their sexual function, but the causality of this relationship needs further investigation.

Educational level has been shown to protect against psychological disorders development in the general population [36, 37]. In line with these results, our study found that educational levels were significantly negatively associated with depression and anxiety in ED patients. This reinforces the findings of existing literature and support the hypothesis that higher levels of education can be a protective factor against depression and anxiety in ED patients. This finding can be attributed to the fact that education precedes and influences other socio-economic indicators, such as family status and income [37], and can make patients more receptive to disease status or understand its expected therapeutic outcomes.

Although age can influence anxiety and depression, this varies with the content. For example, prevalence of depression and anxiety increases with age under conflict settings [38], while the severity of depression and anxiety decreases with age among breast cancer patients [39]. Our data demonstrated that younger age was associated with higher levels of anxiety symptoms. Younger patients find it more difficulty to cope with ED, and may be more anxious due to their multiple social roles and sensitive family roles. Therefore, the psychological burden is heavier among young ED patients.

Furthermore, emerging evidence from previous studies has linked the onset and symptoms of mental disorders in the general population to healthy “lifestyle factors”, which refers to healthy behaviors such as physical activity, diet, smoking, and sleep [40]. For example, a meta-review showed that smoking was a causal factor contributing to the occurrence of severe and moderate mental illness. Moreover, the use of physical activity in specific situations and poor sleep quality were risk factor for mental illness [41]. In our study, we found that healthy lifestyles such as non-smoking, regular sleep, and regular exercise can reduce anxiety or depression symptoms, but anxiety or depression is not always affected by the same factors, with regular sleep only affects anxiety and regular exercise only associated with depression. This may be attributed to the characteristics of ED population or to different mechanisms of anxiety and depression. Anxiety is characterized by worry, feelings of apprehension, and prominent tension, while depression is manifested as slow thinking, depressive mood, and loss of interest [42]. Moreover, although regular sexual partner, treatment history, occupation, hobbies, and coffee drinking were statistically significant in univariate analysis, and a previous study showed that partnered with women with female sexual dysfunction can increase risk of erectile dysfunction [43], they were not in multivariate analysis, thus further research is needed.

Our current findings are important today, it provides insights into the associated factors and prevalence of anxiety and depression, as well as coping strategies among ED patients. Anxiety and depression among patients with ED represent a major public health problem, and a large number of ED patients have both diseases. For ED patients with anxiety or depression, psychosocial interventions including cognitive and behavioral therapy, and different modalities (e.g., marital therapy, sexual skills training, psychosexual education) are recommended (2). In addition, counselling for partner is also recommended [43]. In clinical practice, most Chinese doctors lack the psychological disease diagnosis and treatment background and do not evaluate psychological situation before treatment, which may affect the treatment outcomes of ED. Therefore, in-depth analysis of the psychological state of ED patients and its associated factors may help to explore the association of depression and anxiety with ED to develop individualized treatment options.

### Limitations

First, since this is a cross-sectional design, it was difficult to infer the causality between ED and psychological diseases. Second, all participants were recruited from the men’s outpatient department, and most of the patients were from Hunan province and a few from other provinces, so ample biasness was inevitable, and multicenter or large sample study is necessary. Third, our study lacks patients with severe anxiety and depression, this could be because people with severe symptoms cannot complete the questionnaire independently. Therefore, future studies should be conducted with large sample size.

## Conclusions

In summary, this study demonstrated the prevalence and associated factors of anxiety and depression among patients with ED in China. The prevalence of anxiety and depression in ED patients is high, and the severity of ED, education level, age, smoking, onset time, regular sleep, and exercise were associated with anxiety or depression symptoms. Clinicians should not ignore the psychological problems of ED patients, and they should work more closely with psychiatrists to help patients find reversible risk factors such as unhealthy “lifestyle factors” and administer individualized treatment in a timely manner.

## Data Availability

The datasets generated and analyzed during the current study are not publicly available due individual privacy but are available from the corresponding author on reasonable request.

## Abbreviations

ED: Erectile dysfunction
IIEF-5: 5-Item international index of erectile function
SAS: Self-rating anxiety scale
SDS: Self-rating depression scale
BMI: Body mass index
PASS: Power analysis and sample size
IRB: Institutional review board
SPSS: Statistical product service solutions
SD: Standard deviation
ANOVA: Analysis of variance
